# Crystal Chemistry of High-Temperature Borates

**DOI:** 10.3390/molecules25102450

**Published:** 2020-05-25

**Authors:** Nikolay I. Leonyuk, Victor V. Maltsev, Elena A. Volkova

**Affiliations:** Department of Crystallography and Crystal Chemistry, Lomonosov Moscow State University, 119991 Moscow, Russia; leon@geol.msu.ru (N.I.L.); maltsev@geol.msu.ru (V.V.M.)

**Keywords:** high-temperature borates, anhydrous borates, crystal chemistry, fundamental structural units, boron-oxygen radicals

## Abstract

In recent years borate-based crystals has attracted substantial interest among the research community. The overall importance of this family of materials is reflected in miscellaneous articles and several reviews that have been published over the years. Crystalline borate materials exhibit numerous interesting physical properties, which make them promising for further practical applications. Diversity of functional characteristics results from their high structural flexibility caused in the linkage of planar/non–planar BO_3_ groups and BO_4_ tetrahedra, which can occur as isolated or condensed structural units. This report is a brief review on crystal chemistry and structure features of anhydrous/high-temperature borates. Polymorphism of boron-oxygen radicals has been considered basing on cations’ nature and synthesis conditions. Analysis of the laws governing borates structures and general principles of their systematics was discussed. As a result, an alternative classification of anhydrous compounds has been considered. It is based on four orders of their subdivision: (1) by the variety of anion formers, (2) by the cation charge, (3) by the N = N*_M_*:N*_B_*, i.e., ratio of metal atoms number to the ratio of boron atoms number (N-factor) value indicating the borate structural type (if it is known), (4) by the cation type and size.

## 1. Introduction

A critical analysis of the existing literature data on melting diagrams of borate systems, hydrothermal synthesis and mineralogical field studies indicates that more than 1300 high-temperature (anhydrous) borates and their structural derivatives have been synthesized and also found in Nature [1]. Their crystal structures and structural types have been studied using X-ray diffraction methods. Most of them belong to orthorhombic, monoclinic or trigonal/hexagonal systems. As a rule, the symmetry of borate materials decreases with decreasing cation and the ratio between the number of metal and boron atoms in the compound [2].

**Figure 1:**
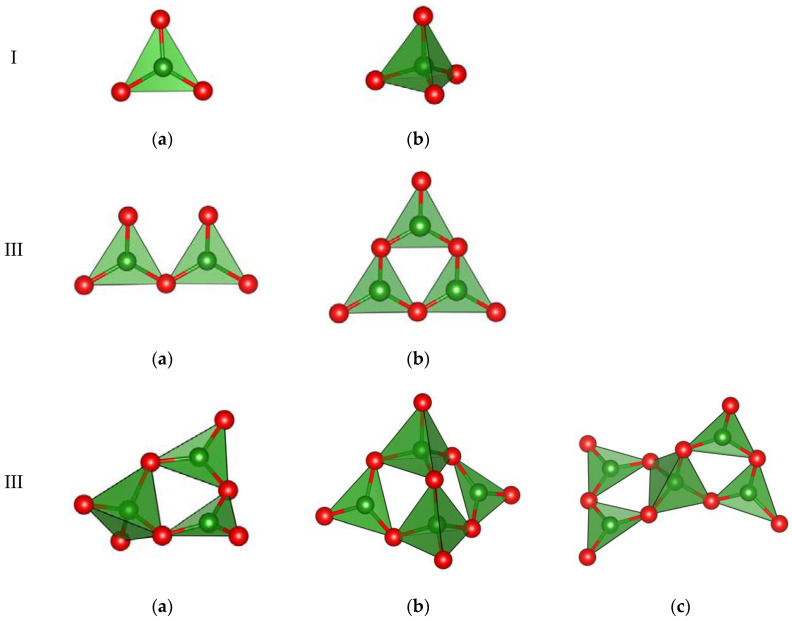
Examples of “building units” for anions and polyanions in anhydrous borates: (**I**) isolated fundamental units, only in orthoborates (**a**), (**b**); (**II**) island clusters, in pyroborates (**a**) and metaborates (**b**); (**III**) combined basic structural units in highly condensed polyanions (**a**), (**b**), (**c**). The green and red balls represent B, and O atoms, respectively.

Isolated BO_3_ triangles predominate in the structures of anhydrous borates, especially, those of binary and more complex compounds (about 65%) [3]. Among the remaining borates, almost one-half are represented by framework structures containing three-dimensional boron-oxygen polyanions (exclusively monocationic compounds), followed by insular layered and chain structures, i.e., those with dispersed individual elements (pyrogroups of two BO_3_–triangles, rings of three triangles, groups of two BO_4_–tetrahedra). In these cases, BO_3_– and BO_4_– groups are joined by sharing common O atoms. Polyanions of compounds with cations of alkali and alkali-earth metals are most diverse. Then, it is followed by isolated (pyroborates and ring metaborates), network and chain-forming structures. There are about fifty types of boron-oxygen anions and polyanions proper (including the BF_4_ group) and a dozen of borosilicate, boroaluminate and boroberyllate anions (Figure 1).

As shown by our experimental studies [3,4], the regularities of the crystallization of borates with trivalent metals are in many respects determined by the specificity of their structure and the composition of crystallization medium. An increase in the content of anhydrous boric oxide in the melts facilitates the synthesis of borates with a higher polymerization of boron-oxygen radicals [2,3,4]. It should be also noted the tendency of boron atoms to tetrahedral coordination, and BO_3_–and BO_4_–groups-to polycondensation, when they prevail over other cations in the structures of compounds synthetized. Similar trends were also observed for some other groups of borates [5,6,7]. It is, therefore, worth assessing the extent to which the above peculiarities are typical for anhydrous borates in general.

## 2. Hierarchy of Basic Structural Clusters

This section provides a concise and precise description of the experimental results, their interpretation as well as the experimental conclusions that can be drawn. In polymeric boron-oxygen constructions of different composition one can easily identify, except for BO_3_–triangles (∆) and BO_4_–tetrahedra (t), comparatively small groups formed by them, most of which are represented in many structures. First this clusters (radicals), but only isolated, were found in anhydrous potassium and sodium metaborates [8,9], and then in three-dimensional frameworks as well [10]. These comparatively small structural components (usually single or double rings), containing 2–5 triangles and tetrahedra, are designated below as combined basic structural units *CSU* in contradistinction to the fundamental (elementary) structural units (*FSU*), i.e., BO_3_–triangles and BO_4_–tetrahedra.

In polyanions of anhydrous borates, it is expedient to discern one more type of structural units. Structural units of this category are, as a rule, more complex and characterize the structures and the structural types of compounds. It is appropriate to designate them, as complete radicals of polyanions (*CRP*). *CRP* can contain more than one *CSU* and/or additional triangles and tetrahedra. Thus, it represents a full repeating fragment of a polyanion (made up of 2–9 *FSU*) corresponding in composition or multiple of the boron-oxygen anion part in the structural formula of the compound.

### 2.1. Fundamental Structural Units

**Figure 2:**
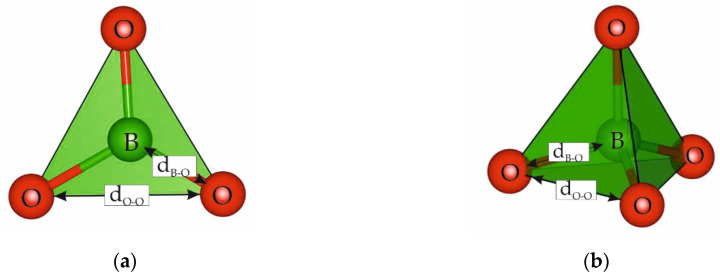
*FSU* in orthoborate structures: (**a**) isolated BO_3_–triangles and (**b**) BO_4_–tetrahedra.

The B-O bond lengths in *FSU* vary within considerable limits, the range of variation being greater in complex structures. Their average values in triangles and tetrahedral amount to 1.37 Å and 1.48 Å, respectively. These values for O-O distances in the sides of triangles equal to 2.38 Å, and in the edges of tetrahedral they are 2.43 Å [1,5] (Figure 2).

### 2.2. Combined Structural Units

**Figure 3:**
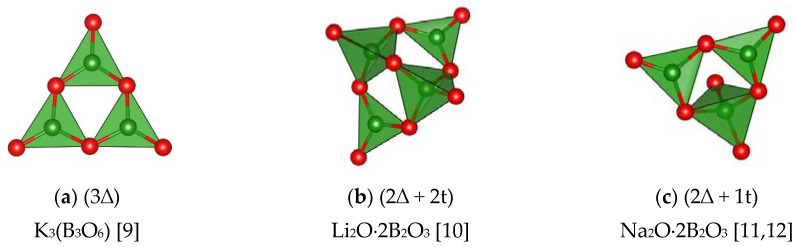

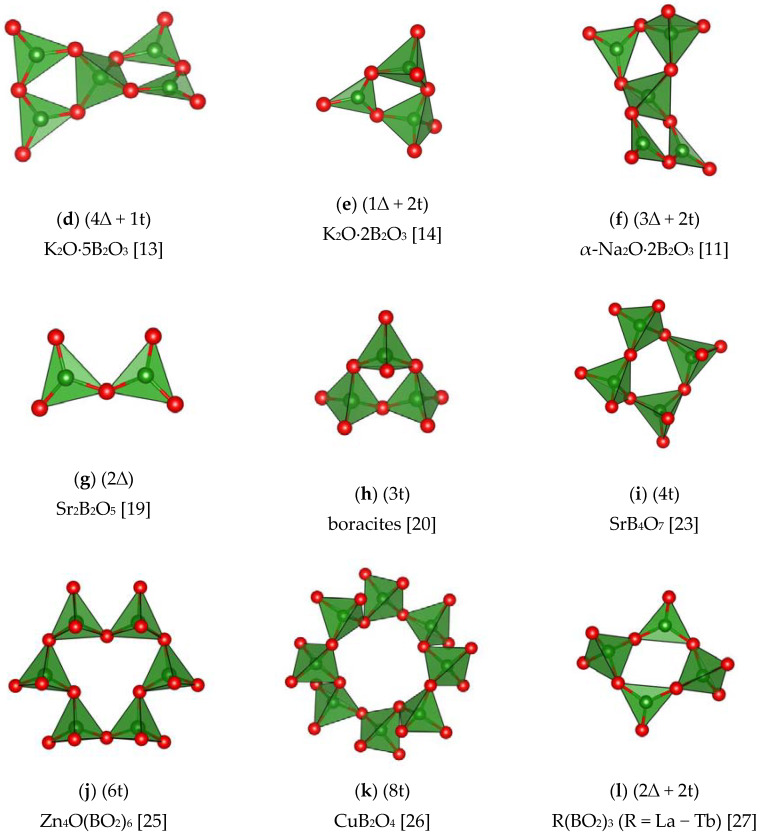
Examples of *CSU* in mono-, di- and trivalent metal simple (monocationic) borates (the green and red balls represent B, and O atoms, respectively). (**a**) K_3_(B_3_O_6_) [9]; **(b)** Li_2_O⋅2B_2_O_3_ [10]; (**c**) Na_2_O⋅2B_2_O_3_ [11,12]; (**d**) K_2_O⋅5B_2_O_3_ [13]; (**e**) K_2_O⋅2B_2_O_3_ [14]; (**f**) α-Na_2_O⋅2B_2_O_3_ [11]; (**g**) Sr_2_B_2_O_5_ [19]; (**h**) boracites [20]; (**i**) SrB_4_O_7_ [23]; (**j**) Zn_4_O(BO_2_)_6_ [25]; (**k**) CuB_2_O_4_ [26]; (**l**) R(BO_2_)_3_ (R = La − Tb) [27]

The structures of alkali metal anhydrous monocationic borates contain a rather limited number of *CSU* of the same kind and their different combinations. In this framework, layered and isolated borate structures of this group most often are encountered single or paired triple rings of triangles and tetrahedra: 1) 3∆, 2) 2∆ + 2t, 3) 2∆ + 1t and 4) 4∆ + 1t (Figure 3a–d), sometimes, they are variously mentioned in the literature as boroxol, diborate, triborate and pentaborate groups, respectively [9,10,11,12,13]. Occasionally, in framework K_2_O·2B_2_O_3_ [14] and layered α-Na_2_O·2B_2_O_3_ [11], were single and double 1∆ + 2t and 3∆ + 2t rings (Figure 3e,f) encountered, not very aptly called ditriborate and dipentaborate groups. This is because the ditriborate group together with the additional BO_3_– triangle in K_2_O·2B_2_O_3_ structure represents configurationally a strongly deformed diborate group.

In borates of divalent metals, namely CaB_2_O_4_, BO_3_–triangles and BO_4_–tetrahedra can form endless chains [15,16,17]. Apart from these, the most borates of divalent and trivalent metals also have other *CSU.* First of all, there is the pyroborate containing isolated pair of BO_3_ triangles [18,19] (Figure 3g). The next one, a ring of three tetrahedra (Figure 3h) has been found in boracite, hausenite and their analogues [20], in CaB_2_O_4_ (III) [21], SrB_2_O_4_ [22], SrB_4_O_7_ and PbB_4_O_7_ [23] structures. The same rings, but without additional *FSU* were discovered in framework CaB_2_O_4_ (IV) [24]. Less widespread are quadruple (SrB_4_O_7_ and PbB_4_O_7_ [23]), sextuple (Zn_4_OB_6_O_12_ [25]) and octuple (CuB_2_O_4_ [26]) rings of tetrahedra (Figure 3i–k). Overall, CSU here are more diversified than in the borates of alkali metals. Among them, the tetrahedral coordination of boron atoms predominates over the triangular one.

In the borates of trivalent metals, *CSU* differing from the others have also been found, e.g., quaternary rings of two BO_3_–triangles and two BO_4_–tetrahedra in corrugated metachains of rare earth borates, *R*(BO_2_)_3_ with *R* = La − Tb [27] (Figure 3l). In such chain, every BO_4_ tetrahedron belongs to two adjacent rings, i.e., the oxygen corners of all the tetrahedra are shared with the triangles, while each of the triangles has one corner unbonded inside the chain. According to [28], isolated triple rings of BO_4_ tetrahedra represent the anionic part of the structure of low-temperature GdBO_3_ and its rare earth analogues. Borate with tetravalent cations are represented by ThB_2_O_5_ and ThB_4_O_8_ compounds. Thorium diborate, ThB_2_O_5_, exhibits α- and β- polymorphs. In the structure of β-ThB_2_O_5_ two corner sharing BO_3_ triangles are linked forming an isolated B_2_O_5_ dimer [29]. Whereas, in the structure of α- modification boron atoms occupy corner sharing BO_4_–tetrahedra and BO_3_–triangles forming 1D zigzag chain [30]. ThB_4_O_8_ structure exhibits infinite chains, which consist of [B_2_O_4_]^5−^ tetrahedral borate groups that are connected through one common oxygen atom [31]. Monocationic borates of pentavalent elements have only isolated BO_4_ tetrahedra [32].

**Figure 4:**
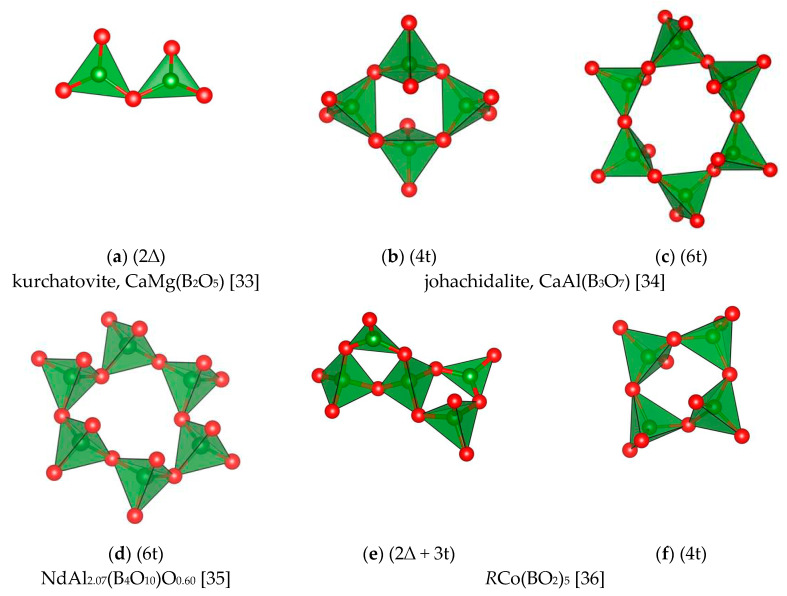
Examples of *CSU* in binary and more complex borates (the green and red balls represent B, and O atoms, respectively). (**a**) kurchatovite, CaMg(B_2_O_5_) [33]; (**b**) and (**c**) johachidalite, CaAl(B_3_O_7_) [34]; (**d**) NdAl_2.07_(B_4_O_10_)O_0.60_ [35]; (**e**) and (**f**) *R*Co(BO_2_)_5_ [36].

The structures of binary and more complex borates predominantly contain isolated BO_3_ triangles, less frequently tetrahedra or pyrogroups (Figure 4a). Only in four types of layered structures, i.e., in jochachidolite, synthetic *R*Co(BO_2_)_5_ with *R* = La − Ho, Na_2_Zn_2_MnB_4.67_O_11_ and *R*Al_2_B_4_O_10.5_ with *R* = La − Nd [33,34,35,36,37], one can identify sextuple, quadruple and binary three-membered rings (Figure 4b–f).

In the *R*-aluminum metaborates synthetized by the authors of [38], the Al atoms are located in five-corner oxygen polyhedra, they can formally be regarded as a transitional compound between the borates proper and aluminoborates. Another type of “transitional” compounds can be represented by A1_5_(BO_3_)O_6_ = Al_2_[Al_3_O_6_BO_3_], in this structure 60% of Al atoms are located in AlO_4_–tetrahedra and 40% of them form AlO_6_–octahedra [39].

**Figure 5:**
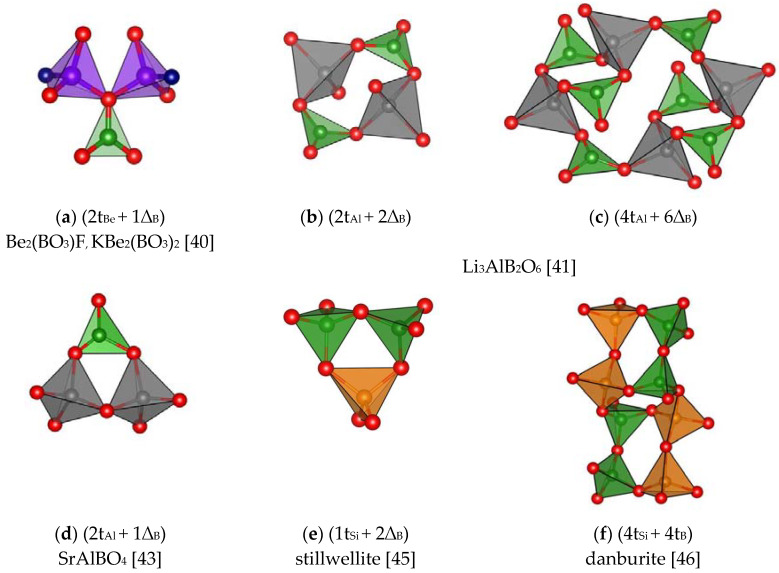
*CSU* in boroberyllates, boroaluminates and borosilicates (the green, red, violet, grey, and brown balls represent B, O, Be, Al, and Si atoms, respectively). (**a**) Be_2_(BO_3_)F_,_ KBe_2_(BO_3_)_2_ [40]; (**b**) and (**c**) Li_3_AlB_2_O_6_ [41]; (**d**) SrAlBO_4_ [43]; (**e**) stillwellite [45];(**f**) danburite [46].

An individual group can be made up of *CSU* (in an overwhelming majority of cases - ring-shaped) in boroberyllates, boroaluminates and borosilicates where Be, Al and Si atoms with a tetrahedral coordination partially substitute boron (Figure 5) (see, for example, Ref. [40,41,42,43,44,45,46]). Unusual illustrations of trivalent Al and divalent Be in the role of boron are found in the structures of CaAlOBO_3_ [42]*,* SrAlBO_4_ [43] and Y_2_AlBeBO_7_ [44]. In the first case, double (2t_Al_ + 2∆_B_) rings in the chains by their configuration resemble the diborate groups very often found in the polyanions of mono- and divalent metal borate structures. The alumoborate metachain, i.e., one-dimensional (1D) chain of [Al_2_B2O8]1D composition in SrAlBO_4_, where (2t_Al_ + 1∆_B_) rings can be identified, is similar to the metaborate chain in calciborite - [B4O8]1D, if the BO_4_ tetrahedra in it are replaced by those of aluminum, i.e., by AlO_4_ groups.

### 2.3. Complete Radicals of Polyanions

**Figure 6:**
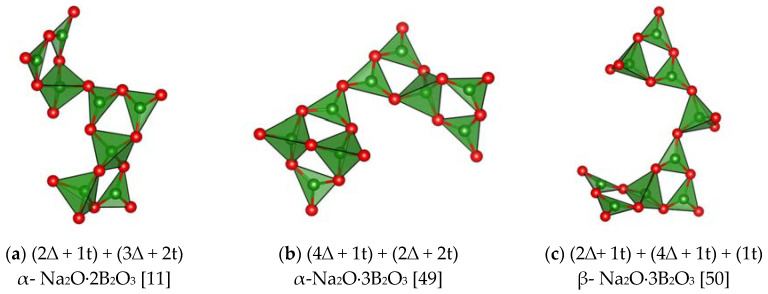

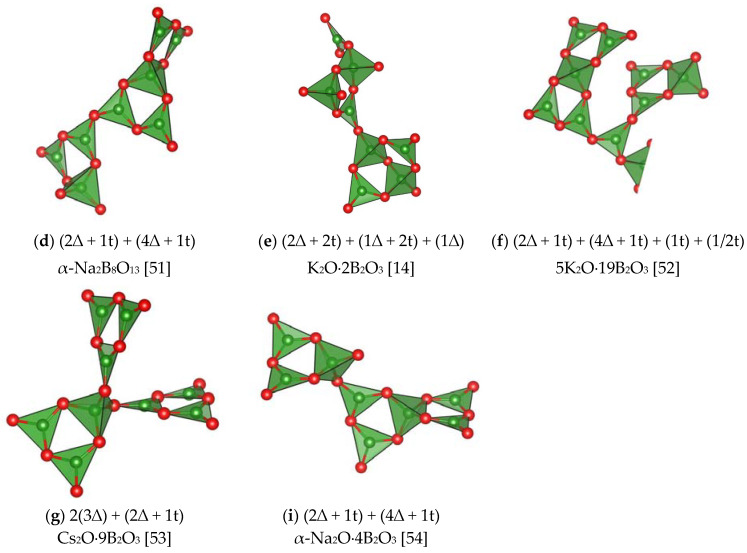
*CRP* in monovalent metal borates which are different from the *FSU* and *CSU* in these compounds (the green and red balls represent B, and O atoms, respectively). (**a**) α- Na_2_O·2B_2_O_3_ [11]; (**b**) α-Na_2_O⋅3B_2_O_3_ [49]; (**c**) β- Na_2_O⋅3B_2_O_3_ [50]; (**d**) α-Na_2_B_8_O_13_ [51]; (**e**) K_2_O⋅2B_2_O_3_ [14]; (**f**) 5K_2_O⋅19B_2_O_3_ [52]; (**g**) Cs_2_O⋅9B_2_O_3_ [53]; (**i**) α-Na_2_O⋅4B_2_O_3_ [54].

**Figure 7:**
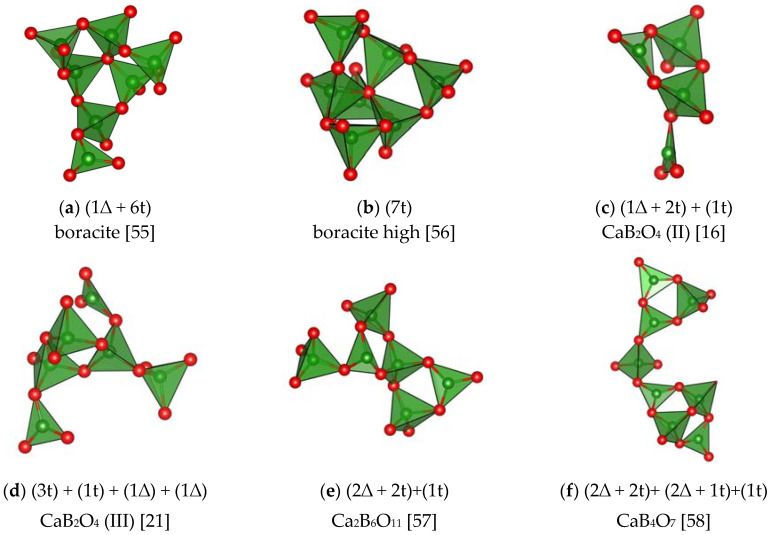

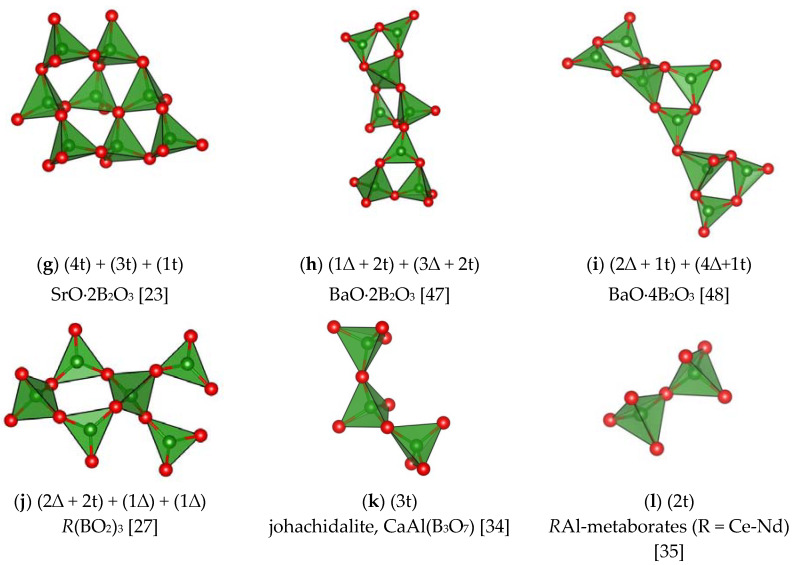
*CRP* in di- and trivalent metal borates different from the *FSU* and *CSU* in these compounds (the green and red balls represent B, and O atoms, respectively). (**a**) boracite [55]; (**b**) boracite high [56]; (**c**) CaB_2_O_4_ (II) [16]; (**d**) CaB_2_O_4_ (III) [21]; (**e**) Ca_2_B_6_O_11_ [57]; (**f**) CaB_4_O_7_ [58]; (**g**) SrO⋅2B_2_O_3_ [23]; (**h**) BaO⋅2B_2_O_3_ [47]; (**i**) BaO⋅4B_2_O_3_ [48]; (**j**) *R*(BO_2_)_3_ [27]; (**k**) johachidalite, CaAl(B_3_O_7_) [34]; (**l**) *R*Al-metaborates (R = Ce − Nd) [35].

**Figure 8:**
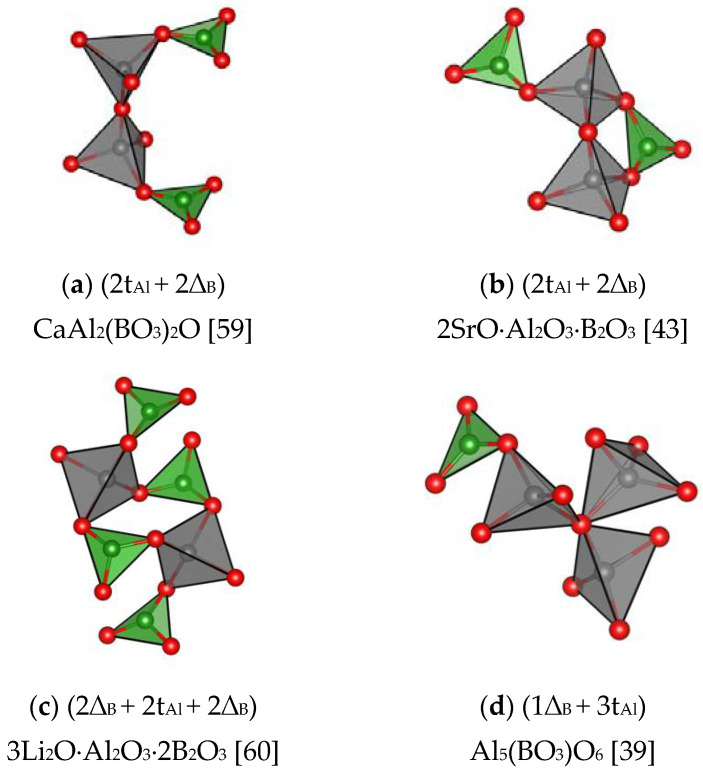
*CRP* in boroberyllates, boroaluminates and borosilicates different from the *FSU* and *CSU* in these compounds (the green, grey, and red balls represent B, Al and, O atoms, respectively). (**a**) CaAl_2_(BO_3_)_2_O [59]; (**b**) 2SrO⋅Al_2_O_3_⋅B_2_O_3_ [43]; (**c**) 3Li_2_O⋅Al_2_O_3_⋅2B_2_O_3_ [60];(**d**) Al_5_(BO_3_)O_6_ [39].

*CRP* in the structures of anhydrous borates are similar to some *CSU* shown in Figure 3 and Figure 4 and they are identical with *FSU* in orthocompounds. The *CRP* have a comparatively complex structure in monovalent metal borates, for example, in boracite, as well as in a number of CaB_2_O_4_ modifications [16,17,21], SrB_2_O_4_, Ca_2_B_6_O_l1_, CaB_4_O_7_, BaO·2B_2_O_3_, BaO·4B_2_O_3_ [23,47,48] (Figure 6 Figure 7 and Figure 8) and in some others. A peculiar *CRP* is found in aluminum orthotriborate, A1_5_(BO_3_)O_6_ [39] or, to be more precise, in aluminum-alumoborate Al_2_(AlO_2_)_3_BO_3_.

Thus, despite the great diversity of structural types of anhydrous borates, most of them contain only three configurations of the basic structural units: *FSU*, *CSU*, and *CRP* (Figure 6, Figure 7 and Figure 8)

## 3. Polymerization of Boron-Oxygen Radicals

Polymerization degree of B_n_O_m_ anions can be defined by the ratio of N = N*_M_*:N_B_ (called as N factor), i.e., by the relation between the number of atoms of a metal (metals, *M*) and B atoms in the formula of a compound. Generally it is equivalent to the N’ = N*_M_*_xOy_:N_B2O3_ ratio. For monocationic borates with cations of odd valence, N = N’, but for those with even valence N = N’/2. The effect of the magnitude of N–factor on the type of boron-oxygen radicals and the degree of radicals polymerization should be analyzed for compounds with the same cations, i.e., separately for Li, Na, K, etc.

The most comprehensive X-ray diffraction data have been accumulated on alkali metal borates. A decrease in the value of N in these compounds leads polyanion complication and changing of structural type. For Li borates, obtained at normal pressure, isolated BO_3_–triangles (*N* = 3) in *α*-Li_3_BO_3_ [61] transform to one-dimensional chains of triangles (*N* = 1) in LiBO_2_ [62], then to three-dimensional chains of (4∆ + 3t) groups (*N* = 4/7) [44] or (2∆ + 2t) groups for *N* = 1/2 in Li_2_O·2B_2_O_3_ [10]. In Na borates isolated groups of 3∆ at *N* = 1 in Na_3_(B_3_O_6_) [8] transform into two-dimensional nets of dipentaborate or triborate clusters with *n* = n_∆_:n_t_ = 5/3 (*N* = 1/2) in Na_2_O·2B_2_O_3_ [11]. Then one can observed transformation to: 1) a double two-dimensional net of pentaborate, triborate rings with additional tetrahedra (*n* = 2) in a metastable low-temperature modification or 2) twinned three-dimensional framework of penta- and diborate groups with the same ratio of BO_3_–triangles and BO_4_–tetrahedra in the stable high-temperature form for *N* = 1/3 in *α*-Na_2_O·3B_2_O_3_ and β-Na_2_O·3B_2_O_3_ [49,50]. Finally, twinned three-dimensional network of penta- and triborate rings with *n* = 3 at *N* = 1/4 are formed in [54]. For potassium borates isolated rings of 3∆ (*N* = 1) in K_3_B_3_O_6_ [9,63] change over to three-dimensional networks: a) of diborate, ditriborate groups having additional BO_3_–triangles with *n* = 1 at *N* = 1/2 in K_2_O·2B_2_O_3_ [14]; b) of penta-, triborate rings, additional BO_3_–triangles and BO_4_–tetrahedra with *n* = 14/5 at *N* = 5/19 in 5K_2_O·19B_2_O_3_ [52]; or c) of pentaborate *CSU* with *n* = 4 (*N* = 1/5) in K_2_O·5B_2_O_3_ [13]. In rubidium and cesium borates isolated rings of 3∆ (*N* = 1) transform into a three-dimensional framework of triborate groups with *n* = 2 (*N* = 1/3) in Cs_2_O·3B_2_O_3_ [12], then into twinned two-dimensional network of triborate and boroxol rings with *n* = 8 (*N* = 1/9) [64]. In the Ag-tetraborate AgO·4B_2_O_3_ the polyanion structure is similar to potassium one [65].

No isolated BO_4_ groups have been found in monovalent metal borates. Isolated BF_4_ tetrahedra are known only in the structures of fluoroborates NaBF_4_ and NH_4_BF_4_ with *N* = 1 [66,67]. The maximal fraction of BO_4_–tetrahedra (*n* = 1) is found in the framework lithium (Li_2_O·2B_2_O_3_) and potassium (K_2_O·2B_2_O_3_) borate structures with *N* = 1/2 but 1/*n* = 3/5 [10,14], and in cesium borate Cs_2_O·3B_2_O_3_ it is even smaller (1/*n* = 1/2) and shifts to *N* = 1/3 [12]. No clear-cut relationship was established between the value of n, the type of BO radical and the degree of the deformation of BO_3_–triangles and BO_4_–tetrahedra, with the exception that isolated *FSU*s are more regular.

With a decrease in the N number in borates of divalent metals, the same tendency is observed as in monovalent metal borates. At *N* ≥ 3/2 (i.e., N*_M_*_O_:N_B2O3_ ≥ 3), only isolated BO_3_–triangles are observed in the structures of monocationic and binary compounds. In pyroborates (*N* = 1), including binary *M*^2+^*M*^3+^B_2_O_5_ compounds, anions are represented by isolated B_2_O_5_ pyrogroups. Metaborate anions (*N* = 1/2) are more condensed: a) infinite metachains of BO_3_–triangles (CaB_2_O_4_-I) [17], b) cyclic groups of three triangles bonded at common corners (BaO·B_2_O_3_ [68], c) ring-shaped three-membered rings of BO_4_–tetrahedra in CuB_2_O_4_ [26]. Thus, in divalent metal metaborates besides BO_3_–triangles, BO_4_–tetrahedra also appear under normal pressure.

Compounds with an even smaller N–factor exhibit only 3D frameworks. The amount of triangular and tetrahedral boron in the structures with *N* = 1/4 is the same, with the exception of SrB_4_O_7_ and PbB_4_O_7_ in which the three-dimensional anionic framework consists only of BO_4_–tetrahedra [23]. In the three-dimensional boron-oxygen net of BaB_8_O_13_ (*N* = 1/8) triangularly coordinated boron (*n* = 3) predominates [48]. Although no clear-cut regularity is observed in the variation of the limits of B-O interatomic distances depending on the N value in divalent metal borates; they are more isometric in orthoborates.

In the structures of borates with trivalent metals at *N* ≥ 1, only isolated BO_3_–triangles (or isolated BO_4_–tetrahedra in Fe_3_BO_6_ [69] and sinhalite MgAlBO_4_ [70]) have been found. In simple *R* metaborates (*N* = 1/3), the polyanions are chain-like with *n* = 2. Simultaneously, in binary borates of tri- and divalent metals, for example, johachidolite CaAlB_3_O_7_ [34], synthetic *R*Co(BO_2_)_5_ [71] and *R*Al_2_B_4_O_10.5_ [35] the polyanions are represented by layers of BO_4_–tetrahedra or BO_4_–tetrahedra together with BO_3_–triangles, even if the N value is equal to 2/3, 2/5 and 3/4, respectively. Most likely, the nature of metals plays a significant role here. Both simple and binary pentavalent metal borates are not numerous and are not distinguished by any special diversity in the anion structure. For monocationic compounds, tetrahedral configuration of boron is preferable, but for binary compounds, it is triangular.

Decrease of the N ratio in borosilicates (in this case, it implies the ratio of the sum of metals to the sum of B and Si atoms) results to increasing of polymerization of the silicon-boron-oxygen motif. Thus, in the structure of grandidierite (Mg,Fe)Al_3_SiBO_9_ [72] (*N* = 2), the anion consists of isolated BO_3_–triangles and SiO_4_–tetrahedra, but in stillwellite LaBSiO_5_ (*N* = 1/2), it is represented by BO_4_–metachain encrusted with discrete SiO_4_–tetrahedra [45]. In danburite CaB_2_Si_2_O_8_ (*N* = 1/4) a silicon-boron-oxygen framework of Si_2_O_7_– and B_2_O_7_–diorthogroups is observed [46]. If the sum of metals exceeds the total amount of Si and B atoms, the boron is coordinated by three oxygen atoms. Taking into account the stillwellite structure, boron-oxygen *FSU* are more predisposed to polymerization in comparison with silicon-oxygen ones. It is noteworthy that there are practically no minerals of anhydrous borosilicates with the amount of boron predominating over the silicon content (with the exception of cappelenite BaY_6_(Si_3_B_6_O_24_)F_2_ [73]), whereas borosilicates enriched with silicon are quite numerous. The shortage of adequately interpreted structures for boroaluminates (with the exception of synthetic A1_5_(BO_3_)O_6_ [39]) makes it impossible to disclose their regularities. Even smaller amount of structural data are published for boroberyllates.

Coordination metal-oxygen polyhedra is more regular in structures with large N value, i.e., when they constitute the base of these structures. At small N value, not numerous metal atoms adapt themselves to the anionic motif. In other words, coordination oxygen polyhedra of metals in highly polymerized borates seem to be mostly determined by the nature of free spaces in the boron-oxygen base, but not by the directed bonds.

Among the borates having high N–factor values, the tendency to anion polymerization increases in compounds with smaller cations. For example, the difference in B-O interatomic distances inside the ring of potassium metaborate K_3_B_3_O_6_ [63] is smaller than in sodium compound Na_3_(B_3_O_6_) [8]. However, in lithium analogue LiBO_2_ [62], the ring already becomes energetically disadvantageous orates. In the meantime, for highly condensed compounds the tendency of B-O anions to polymerize somewhat increases with an increase in the cation sizes.

An increase in the cation charge facilitates the weakening of the polymerization of BO_3_–triangles and BO_4_–tetrahedra and makes the tetrahedral coordination of boron atoms more preferable. In borates with comparatively weak singly charged and large divalent cations, the stability of the structure is ensured by the delocalization of anion charge, i.e., by a decrease in its formal specific charge during the polymerization process. In the case of cations with relatively high charge, which capable to form around themselves strong coordination polyhedral, making up the base of the structures, and the boron-oxygen anion stability is no longer of decisive importance. In such structures, therefore, not only isolated BO_3_–triangles are commonly widespread, but BO_4_–tetrahedra with an even higher negative charge (−5) as well.

## 4. Polymorphism of B_n_O_m_ Polyanions

Metaborate radical of [BO_2_]*_n_* composition, mostly widespread in polyborates, was found in isolated (0D), chain (1D), ribbon (1D), layered (2D) and three-dimensional (3D) borates (Table 1). Index *n* in the formula [BO_2_]*_n_* for the currently known varieties of polyanions in metaborates assumes all the values from 1 to 6. With increasing polymerization of this metaradical, the triangular coordination of boron atoms regularly changes into BO_4_–tetrahedra as one passes from the insular to chain, layered and 3D structures.

Pressure significantly affects the formation of metaborates’ structural motifs. Thus, in lithium borate obtained at low pressure and temperature, the boron atoms are located in oxygen triangles that are condensed into chains [62], but γ-LiBO_2_ crystals synthesized at 950 °C and a pressure of 15 kbar have 3D structures with tetrahedral coordination of boron atoms [74,75]. In the Ca metaborate structure, stable at normal temperature, i.e., CaB_2_O_4_ (I), B and Ca atoms have triangular and eightfold coordination, respectively [17]. With the transition of this modification into CaB_2_O_4_ (II) synthetized at 12–15 kbar, half of the boron atoms increase their coordination number to four [16]. In CaB_2_O_4_ (III), which can be obtained at 900 °C and 15–25 kbar pressure, the fraction of boron atoms that preserve their triangular coordination is already only 1/3 [21]. In this case, for one-third of Ca atoms the coordination number increases to ten. In the structure of CaB_2_O_4_ (IV) all the B atoms are located in oxygen tetrahedra, and the coordination number of Ca increases to 9–12 [24]. A similar situation was observed in Sr metaborate [23,76].

A distinguishing feature of [B_4_O_7_]*_n_* polyanions (*n* = 1 and 2) is their high degree of polymerization. Only one of them, α-Na_2_O·2B_2_O_3_ is layered, in all others representatives were found 3D anionic motifs (Table 2). In all borates having [B_4_O_7_]*_n_* radicals, with the exception of α-Na_2_O·2B_2_O_3_, SrO·2B_2_O_3_ and PbO·2B_2_O_3_, the anions contain equal amounts of triangular and tetrahedral boron-oxygen coordination. The ratio of n_∆_: n_t_ = 5/3 in α-Na_2_O·2B_2_O_3_ is accompanied by part of BO_3_ triangles being the corners unshared with other *FSU*, and in the exclusively tetrahedral frameworks of SrO·2B_2_O_3_ and PbO·2B_2_O_3_, part of oxygen atoms is coordinated with three boron atoms [23].

The 3D polyanion of [B_4_O_7_]*_n_* composition in borates with comparatively small cations is made up of 2∆ + 2t diborate groups only. For larger M^2+^ cations the architectural principle was found in the twinning of 3D boron-oxygen nets, designated in Table 2 by doubling the contents of the square brackets. An increase in cation size leads to a strong deformation of diborate groups in K_2_O·2B_2_O_3_. In CaO·2B_2_O_3_ triborate rings with additional tetrahedra are already stable, but a low-symmetry 3D net made up of dipentaborate and ditriborate *CSU* [47,76] is represented in the borate with the largest cation, barium.

The third and the fourth most widespread boron-oxygen clusters are B_3_O_5_ and B_8_O_13_, respectively (Table 3). The first one takes part in the formation of layered and 3D structures, and the second was found in 3D borates only. Common features of structures with these two radicals are their comparative complexity, predominance of BO_3_–triangles over BO_4_–tetrahedra, low symmetry and the twinning of boron-oxygen nets. On the whole, with an increase in the ratio of the total number of boron atoms to that of oxygen atoms in the polyanions their composition and structure become more complex.

## 5. Isostructural Series

Isostructural series are widely encountered among high-temperature borates. Two dozen borates *M*^2+^*M*^3+^[BO_3_]O_3_ with warwickite-type structure and a considerable range of *M*^2+^:*M*^3+^ ratio are limited both by the sizes of trivalent cations close to Al^3+^ and lanthanides [77,78]. In this case, Ca^2+^ has the maximum radius value among *M*^2+^cations.

Isostructural with respect to one another are numerous borates of the ludwigite-vonsenite group, *M*^2+^Fe^3+^[BO_3_]O_3_, where *M* = Mg, Fe, Cu, Co, Ni, partially Sn [77]. This structure is also preserved in Co compounds, where Fe^3+^ is substituted by Cr, Ga, V, Sc, as well as in Fe^2+^ borate [78]. Trivalent iron is almost half substituted by aluminium in aluminoludwigite. However, the substitution of Fe^3+^ by Mn^3+^ in pinakiolite and orthopinakiolite, as well as a substantial inclusion of Sn^4+^ in hulsite, cause a considerable reduction of the symmetry of the structures [79].

Another group of di- and tetravalent metal borates and binary borates of trivalent elements (is headed by nordenskioldine CaSn[BO_3_]_2_ [80] which is isostructural to dolomite. More than fifty compounds compose the boracite group [56], with six minerals among them: boracite, stassfurtite, gauesinite, congolite, ericaite and chambersite. It is not only with various divalent cations anhydrous boracites were synthesized, but with chromium and lithium as well. One can also mention here the isostructural groups of *M*B_4_O_7_, where *M* = Mg, Mn, Zn, Cd; *M*_2_B_2_O_5_ with *M* = Mg, Mn, Fe, Co, Cd; and *M*_3_B_3_O_6_ (*M* = Na, K, Rb, Cs) in which the size of cations differs considerably.

Structural peculiarities of rare-earth borates should be specifically noted. Analysis shown that isostructural along the whole *R* series are only those compounds where the mutual linkage of these cations is comparatively small.

Orthoborates with the general formula *M*^3+^BO_3_ can be structurally subdivided into three groups: (1) isostructural to calcite; (2) isostructural to aragonite; (3) compounds with a structure close to the third modification of CaCO_3_–vaterite. Of them only LuBO_3_, or, to be more specific, its low-temperature modification [81], belongs to the first group. The size of trivalent cation and the *c*:*a* ratio in it are obviously close to the maximum limiting values at which borates with the structure of calcite are stable (these values seem to be minimal in AlBO_3_ [82], synthesized only at high pressures [83]). It is thus seen that in monocationic orthoborates (*N* = 1) the characteristics of each *R* ion (electron shell structure, its radius) exert a decisive effect on the formation of the structure.

In *R*(BO_2_)_3_ metaborates, although the influence of *R* is less pronounced here, the replacement of cations by smaller ones still leads to the deformation of B-O metachains. Therefore, two structural types for them are known. The situation is approximately the same with *R*Co(BO_2_)_5_ metaborates.

Double orthoborates of the *R*_2_Sr_3_(BO_3_)_4_, *R*_2_Ba_3_(BO_3_)_4_ and *R*_2_Ca_3_(BO_3_)_4_ families are already isostructural with one another, although in some compounds the intensities of X-ray reflection do not coincide [84].

In *RM*_3_(BO_3_)_4_ (*M* = Al, Ga, Sc, Fe, Cr) borates with the huntite-type structure, the *R-*oxygen polyhedra are essentially simplified and constitute trigonal prisms deformed to a different extent. In these structures, *R*O_6_–polyhedra are isolated from each other, and the base of the motif is formed by columns of Al, Ga, Sc, Fe, or Cr octahedra connected by isolated BO_3_–triangles [85,86,87,88,89]. When Al is substituted by Fe, and then by Ga, in the Nd*M*_3_(BO_3_)_4_ compound, the symmetry of *M*O_6_–octahedron is increased, with some increase in the size of *R*O_6_–prisms. The relative sharing of *R* ions is not great here, and their specific properties tell even less on the formation of the structure. It seems that in this family of double trivalent metal borates the critical values of the ratio of cation sizes are close to Al^3+^/Nd^3+^ on the one side and to Fe^3+^/Sc^3+^ on the other. Also, there is monoclinic structural modification of NdAl_3_(BO_3_)_4_. In the case of large *R* cations of the cerium subgroup, layered double *R*Al-metaborates with *N* = 3/4 are also synthesized [38]. Despite out numerous attempts it was impossible to obtain LaAl_3_(BO_3_)_4_, and ScFe_3_(BO_3_)_4_ was also crystallized with difficulty in a narrow range of conditions [90].

## 6. Structural Formulas of Polyborates

In the above text formulas of all anhydrous borates are given in the form taken from the literature, i.e., mainly as a ratio of metal oxide to boron oxide. Although they clearly reflect the N′ ratio, their structural formulas are undoubtedly more informative for polyborates with known crystal structure. Showing the ratio of cations and anions in the compounds, the *CRP* composition structure type (isolated anions, clusters, chains and ribbons, layers, 3D motifs), they can show, if necessary, the polyanion nature (simple or twinned), the amounts of triangularly and tetrahedrally coordinated boron atoms. Since most of the polyanions are built of single and double rings, it is useful to note this in the formula too. It could be provided them even more information, but because of the polyanions complexity, their structural formulas would become too bulky. Table 4 lists both complete and abbreviated structural formulas for some polyborates that have been studied.

The composition of repeating radical is enclosed in square brackets with the 1D, 2D or 3D symbols for various types of structures (one-, two- or three-dimensional polyanion, respectively). In the case of a twinned polyanion, the composition of one of the equivalent boron-oxygen nets is doubled (indicated by the figure of two after the square brackets). The maximum information is contained in the first complete version of the formula, where *CSU* are shown, as well as additional BO_3_–triangles and BO_4_–tetrahedra. The second version, as an alternative for writing out the formula, only shows a relationship between the triangularly and the tetrahedrally coordinated boron. For example, since the three-dimensional polyanion in Li_2_O·2B_2_O_3_ consists of 2_∆_ + 2_t_ diborate groups [10] its chemical composition can be written out as [(B_2_^∆^B_2_^t^O_7_)]_3D_ or in the abbreviated form as [B_4_O_7_]_3D_. The negative charge of one such radical is equal to two. The structural formula will, therefore, have the form of Li_2_[B_4_O_7_]_3D_. In contradistinction to Li_2_[B_4_O_7_], in Mg, Mn, Zn and Cd borates with *N*^′^ = 2, the polyanions consist of twinned three-dimensional boron-oxygen nets. Therefore, their structural formula is *M*_2_^II^[B_4_O_7_]_2(3D)_. In the α-Na_2_O·2B_2_O_3_ structure, the two-dimensional polyanionic net consists of dipentaborate and triborate *CSU*, with the latter each containing a free O atom [11]. Polyanion composition of this sodium borate can be written as (B_3_^∆^B_2_^t^O_8.5_) + (B_2_^∆^B^t^O_5.5_) = [B_5_^∆^B_3_^t^O_14_]_2D_ = [B_8_O_14_]_2D_; its negative charge is equal to four, and borate structural formula will be represented in the form of α-Na_4_[B_8_O_14_]_2D_. In K_2_O·2B_2_O_3_, the composition of three-dimensional polyanionic network, consisting of (2∆ + 2t) diborate and (1∆ + 2t) triborate groups with additional BO_3_ triangles (1∆) [14], is (B_2_^∆^B_2_^t^O_7_) + (B^∆^B_2_^t^O_5.5_) + (B^t^O_1.5_) = [B_4_^∆^B_4_^t^O_14_]_3D_ = [B_8_O_14_]_3D_. The charge of this polyradical is four, the borate structural formula can be look like K_4_[B_8_O_14_]_3D_.

The anionic motif of metastable β-Na_2_O·3B_2_O_3_ consists of double two-dimensional networks represented by pentaborate (4∆ + 1t), triborate (2∆ + 1t) rings and additional tetrahedra (1t) [50], i.e., polyanion composition is [(B_4_^∆^B^t^O_8_) + (B_2_^∆^B^t^O_5_) + (B^t^O_2_)] × 2 = [B_6_^∆^B_3_^t^O_15_]_2(2D)_ = [B_9_O_15_]_2(2D)_; and structural formula of this borate β-Na_6_[B_9_O_15_]_2(2D)_. The composition of three-dimensional twinned α-Na_2_O·3B_2_O_3_ polyanion made up of triborate (4∆ + 1t) and diborate (2∆ + 2t) *CSU* is the same as that of β-Na_2_O·3B_2_O_3_ [49]: [(B_4_^∆^B^t^O_8_) + (B_2_^∆^B_2_^t^O_7_)] × 2 = [B_6_^∆^B_3_^t^O_15_]_2(3D)_, and Na-triborate structural formula can be written as β-Na_6_[B_9_O_15_]_2(3D)_. Cs_2_O·3B_2_O_3_ has a simpler formula since its three-dimensional boron-oxygen network consists only of triborate groups (2∆ + 1t) [12] which build polymerized radical [B_2_^∆^B^t^O_5_]_3D_ in structural formula of this compound Cs[B_3_O_5_]_3D_.

In the structures of α-Na_2_O·4B_2_O_3_ (and silver borate of similar composition) the binary three-dimensional anionic motif is represented by pentaborate (4∆ + 1t) and tetraborate (2∆ + 2t) *CSU* [54]. Its polymerized radical [(B_4_^∆^B^t^O_8_) + (B_2_^∆^B^t^O_5_)] × 2 = [B_6_^∆^B_2_^t^O_13_]_2(3D)_ = [B_8_O_13_]_2(3D)_ having charge four takes place in borate structural formula α-Na_4_[B_8_O_13_]_2(3D)_. General formula, i.e., gross composition of 5K_2_O·19B_2_O_3_ (K_2_O·3.8B_2_O_3_, which has a similar boron content, a three-dimensional polyanion built of triborate (2∆ + 1t), pentaborate (4∆ + 1t) groups, additional tetrahedra (1/2t) and triangles (1∆) [52] corresponds to K_5_[B_19_O_31_] containing (B_4_^∆^B^t^O_8_) + (B_2_^∆^B^t^O_5_) + (B_0.5_^t^O) + (B^∆^O_1.5_)] × 2 = [B_7_^∆^B_2.5_^t^O_15.5_] × 2 = [B_14_^∆^B_5_^t^O_31_]_3D_ = [B_19_O_31_]_3D_ radical. Structural formulas of all other borates can be derived in a similar way.

## 7. Classification

Any systematic is usually aimed at finding particular regularities in the system studied, in order to predict a variation of its characteristic features. Classification of numerous borates with their specific structural features is far from a simple matter, but in many respects, it implies the progress of their study. The chemical, crystallochemical and genetic systematics undertaken with the accumulation of factual data reflects a considerable extent of knowledge that had been acquired by the moment of classification. Each subsequent classification is, as a rule, superior to the previous one. The schemes proposed by 1966 were thoroughly and critically discussed in the review [91]. Therefore, there is a sense in dwelling shortly on some of them here.

In [92], by analogy with silicates, the following borate subclasses are described: (1) “nesoborates”, i.e., compounds with isolated BO_3_ triangles and BO_4_ tetrahedra, (2) “soroborates” (grouped), (3) “inoborates” (chainlike), (4) “phyloborates” (layered) and (5) “tectoborates” (three-dimensional). Its main shortcoming is the absence of a definite regularity in the systematics of isolated boron-oxygen polyanions, including a great number of other structural units. As a result, it was not possible to ascertain the relations between borates belonging to different groups and to include a number of synthetic borates.

The crystallochemical classification of borates suggested in [91] is based on two main characteristics: the structure of boron-oxygen polyanions and the manner in which they are combined. It also takes into account the peculiarities of the borate structures, consisting in a much greater variety of their polyanions, in comparison with silicates, phosphates, etc. In this classification, boron-oxygen isolated polyanions, as well as chainlike, layered, and 3D motifs are examined in detail. In addition, possible ways of combining anions and polyanions are analyzed and their general formulas are derived. All borates are subdivided into four orders (subclasses): insular, chainlike, layered and three-dimensional.

Insular borates are divided into eight suborders: (1) isolated non-ringed with isolated polyions; (1*a*) non-ringed with dimers of B(O,OH)_3_ triangles and B(O,OH)_4_ tetrahedra; (2) one-ringed triborates with isolated polyions; (3) two-ringed borates with isolated polyions; (4) three-ringed borates; (5) four-ringed borates; (6) borates with mixed polyions and (7) borosilicates. Three-ringed borates have no representatives, only one four-ringed borate was known, as well as one borosilicate. In all the other borosilicates whose structures were interpreted by that time, B and Si atoms constituted the general motif. In suborders (1), (1*a*) and (2), the families of oxygen-containing and hydroxyl borates were singled out, but tetra- and pentaborates were fallen into the third suborder.

Chainlike borates have been subdivided into following categories: (1) non-ringed; (2) one-ringed; (3) two-ringed; (4) three-ringed; (5) borates with mixed polyions and (6) borosilicates. For the fourth and the sixth suborders, there were no representatives, and only two borates were fallen into the fifth suborder. In non-ringed chainlike borates, the families of oxygen-containing and hydroxyl borates (one compound) were identified, and the families of tetra- and pentaborates represent the two-ringed compounds.

Layered and three-dimensional borates are also regarded as having six similar suborders. Three-ringed three-dimensional borates were yet unknown, but non-ringed three-dimensional representatives are divided into oxygen-containing and hydroxyl (only one compound) borates, and the tetra- and pentaborate families are fallen into the two-ringed suborder.

Within most of the suborders and families, the compounds were classified by the basicity of individual polyions (zero-, uni-, di-, tri-, tetra- and pentabasic) in [92].

As distinct from Tennyson’ systematic [93], in [91], borates with isolated BO_3_–triangles and BO_4_–tetrahedra are included into the section of insular borates, as well as borates with isolated “mono”-ions and dimmers. The classification described in Ref. [91] comprises practically all the borates and many borosilicates known at the time, both with interpreted and hypothetical structures, and reflects the regularities of the polymerization of borates (mainly, hydrated ones). A place was found in it for 119 of the more than six hundreds known by that time anhydrous borates and their polymorphic modifications. All of them were mainly attributed to the section of insular borates, a small part was assigned to three-dimensional and to chainlike compounds, but no comment is made on layered borates. Detailed systematics of boron-oxygen radicals can be considered as a progressive step in understanding the crystal chemistry of this unusual class of inorganic polymer compounds.

Nowadays, the number of anhydrous high-temperature borates with interpreted structures and structural types is several times in comparison with the middle of last century. It is, therefore, quite natural that some of them, mainly those with hypothetical and approximately solved structures, after the interpretation or refinement had to be moved from one section of the classification scheme to another. For this reason, for example, barium metaborate with crystal chemical formula of Ba[B_2_O_4_], included in the section of chainlike compounds, a year later proved to be insular [48]. Its structural formula should be written out as Ba_3_[(B_3_O_6_)_2_]. Also, the crystallographic characteristics of Mg[B_2_O_4_] were unknown before to be assigned to the same order on the strength of the data [92]. should be noted that in a later work [76] the authors doubt whether this compound actually exists. The situation is approximately the same with 2PbO⋅B_2_O_3_ appearing in the same group with B_2_O_5_ dimers among insular borates [76].

The subsequent refinements and interpretations of crystal structures have also revealed numerous inaccuracies in the classification of 3D borates. For instance, triborates with the general structural formula *M*^1+^[B_3_O_5_], where *M*^1+^ = Li, Na, K, Rb, as well as Mg[B_2_O_5_]_2_ have been included in the group of ringed three-dimensional compounds. However, the polyanion in β-NaO·3B_2_O_3_ happened to be a twinned layered one made up of pentaborate (4∆ + 1t), triborate (2∆ + 1t) groups and the additional BO_4_ tetrahedra bonding them [50], and therefore its structural formula should be β-Na_6_[B_9_O_15_]_2(2D)_. The three-dimensional α-modification of Na_2_O·3B_2_O_3_, also with a 3D twinned polyanion of diborate (2∆ + 2t) and pentaborate (4∆ + 1t) *CSU* [49], can probably be better described by a similar structural formula α-Na_6_[B_9_O_15_]_2(3D)_. In K-triborate, cell parameters were determined only by that time [92]. For Li- and Rb- borates, no crystallographic data were known then and MgO·3B_2_O_3_ has was not synthesized yet, in our knowledge. Layered sodium borate with *N* = N*_M_*:*N*_B_ = 1/2 and, as established later [11], with the anion of triborate (2∆ + 1t) and diborate (3∆ + 2t) *CSU*, has been placed by the authors of [90] into the section of two-ringed three-dimensional tetraborates with 3D polyanions. However, its more realistic structural formula might be written as α-Na_4_[B_8_O_14_]_2D_. By that time, there were no detailed structural data for some other 3D borates: Li_2_[B_8_O_13_], Na[B_5_O_8_], Cs_2_[B_4_O_7_], Cs_2_[B_8_O_13_], Cs[B_5_O_8_] and α-Cs_2_O·5B_2_O_3_, with exception of their lattice cell parameters [92]. As for K_2_[B_8_O_13_], only the structure of a compound with close composition, 5K_2_O·19B_2_O_3_ (K_2_O·3.8B_2_O_3_) has been solved [52]. Taking into account the X-ray diffraction studies performed later, the crystallochemical formulas of K_2_[B_4_O_7_], Ca[B_4_O_7_], K[B_5_O_8_], Rb[B_5_O_8_], Na_2_[B_8_O_13_], Ag_2_[B_8_O_13_] and Ba_2_[B_8_O_13_] seem to be not so adequate in Ref. [92]. Probably, it would be better to write them as K_4_[B_8_O_14_]_3D_, Ca_2_[B_8_O_14_]_3D_, α- and β-K_2_[B_5_O_8_]_2(3D)_, β-Rb_2_[B_5_O_8_]_2(3D)_, α-Na_4_[B_8_O_13_]_3D_, Ag_4_[B_8_O_13_]_2(3D)_, and Ba[B_8_O_13_]_2(3D)_, in correspondence with the composition of their polyanions. In addition, boracite was regarded as non-ringed, because of the insufficiently accurate interpretation of its structure [94].

Finally, it should be noted that in accordance with [23], the first SrB_4_O_7_ structure determination by the author of [95] was also incorrect. Namely, based on this example it was assumed possible for the polymerization to take place not only by the combining of the corners of BO_3_–triangles and BO_4_–tetrahedra but also the edges of the tetrahedral [91]. In this connection, it is unlikely that approximately interpreted in [7] layered Na_2_Zn_2_MnB_4.67_O_11_ structure with a very close proximity of triple-charged B^3+^ can be stable.

Another attempt to systematize borates (including organic compounds) was undertaken by G. Heller [96]. It was based on the cation type and the number of boron atoms in the polyanion structural unit. This classification schematically presents the possible polyanions and gives several examples of different structures (many of which later proved to be incorrect) set out in accordance with the number of boron atoms in the basic polyanion structural unit and the type of the anionic radical (isolated, chainlike, layered, three-dimensional). An attempt to encompass numerous anhydrous, hydrated borates and organoboron compounds has made it very cumbersome and led to a number of errors and discrepancies, including representation of the crystallochemical formulas of some compounds.

Christ and Clark [6] have proposed a rational crystallochemical classification of the anions of hydrated borates. The authors have identified the basic polyanion structural units, suggested an abbreviated notation, and the rules of their formation. They have also deduced crystallochemical formulas from the structural data at hand, and the other compounds were combined in a separate group. It was assumed that polymerization could be realized in the following schemes: (1) by the corners of BO_3_–triangles and BO_4_–tetrahedra being combined, (2) by elimination of water from isolated boron groups, (3) by complication of anions into additional groups. The most probable sequence was shown for the addition of protons to the oxygen atoms in hydrous borates.

Therefore, every systematics of borates has made a contribution to the development of the crystallochemistry of this class of compounds. New data on borate crystal structures require further refinement and the revision of existing classifications. This primarily concerns numerous anhydrous compounds the peculiarities of whose structure and crystallization have not been analyzed taking into account the latest data.

The major source of errors in all the systematics is associated with underestimating the regularities of the polymerization of boron oxygen anions. That is why some borates are often prematurely placed into certain sections of the classification schemes. Moreover, this is not surprising since in the course of their study a number of characteristic features previously not taken into account became known. For instance, all crystallochemical systematics did not take into consideration that a part of oxygen atoms in highly condensed 2D and 3D polyanions being cannot be not coordinated not only with two, which is usual, but also with one, three and even four boron atoms. All this, as well as a number of other factors, have introduced substantial uncertainties into the classification of compounds with unknown structures.

In order to avoid such ambiguities, borates with unknown structure should not be included in the crystallochemical systematics. On this way, however, one of the main objectives of classification will not be met, i.e., it will not serve as a basis for theoretical and experimental research, will not favor to forecast structures and properties of new materials. In the case of distribution and redistribution of numerous subsequently studied anhydrous borates among the sections of a latest crystallochemical systematics, similar (with the same cations and having close *M*_x_O*_y_*/B_2_O_3_ ratios) compounds and even borates with analogous structure will be placed into unsuitable for them units. This is because only the structure and composition of the anion (polyanion) were taken into account leaving aside the cation type, size and charge. At times it will be difficult to explain the difference between structures with polyanions of similar composition but with different cations, in order to understand the nature of boron-oxygen radicals polymerization, etc. As a result, it seems impossible to expect a tangible assistance from such systematics on the way of interpretation and refinement of structures, and predicting new compounds.

Ways of searching for a more flexible classification can be based on the general regularities in the structure of this class of borate materials, and on knowledge about the previous systematics of borates and other compounds [97]. Analysis of anhydrous borates structure, composition and conditions shows that there is a sense to examine them separately within the framework of the general classification of borates. In addition, this is because of the specificity of their structures. Thus, e.g., Christ and Clark have shown that in every known hydrated borate structure, in contradistinction to anhydrous borates, there is only one type of the basic structural unit in the anion [6]. The difference between them is clearly illustrated by the comparison of Ca_2_B_6_O_11_ structures and the Ca_2_B_6_O_11_·*x*H_2_O series, where 1 ≤ *x* ≤ 15. Isolated or bonded into chains and layers triborate groups of one BO_3_–triangle and two BO_4_–tetrahedra represent anions of all the hydrated Ca borates. In the anhydrous 3D borates, usually regarded as the end member of this series, the paired rings of two BO_3_–triangles and three BO_4_–tetrahedra are bonded into a framework by additional tetrahedra [96]. For this reason G. Christ and J. Clark emphasized the difference in the structure of anhydrous and hydrated borates and suggested the necessity of their independent analysis.

When classifying anhydrous high-temperature borates, therefore, one should bear in mind the set of the following prerequisites, most of which are individually well known:

(1) In crystal structures each boron atom is bonded with three or with four oxygen atoms in BO_3_–triangles and BO_4_–tetrahedra;
(2) In one structure not only triangular or only tetrahedral coordination is possible, but both of them jointly as well;
(3) Isolated BO_3_–triangles and BO_4_–tetrahedra are not found jointly, insular polyanions;
(4) A decrease in the *N* = N*_M_*/N_B_ ratio (N-factor), as well as an increase in cation size (although to a smaller extent), leads to an increase in the degree of polymerization of the anion and raises the *n* = n_∆_/n_t_ number (at *N* ≤ 1), whereas an increase in cation charge causes the inverse tendency;
(5) Polymerization, or the formation of chains, layers and frameworks, is actualizing by the sharing corners of triangles and tetrahedra (the sharing edges has up to now not been proved conclusively);
(6) In 3D and 2D polyanions (less frequently in chainlike and insular ones), BO_3_–triangles and BO_4_–tetrahedra tend to combine into comparatively compact *CSU*, i.e., diborate (2∆ + 2t), triborate (2∆ + 1t), pentaborate (4∆ + 1t), boroxol (3∆), ditriborate (1∆ + 2t), dipentaborate (3∆ + 2t) and other single and double ringed boron-oxygen negative charged polymerized radicals;
(7) Complex polyanions of anhydrous borates of uni- and divalent metals tend to twinning;
(8) In most of complex polyanions, each oxygen atom is bonded with two boron atoms, for such compounds as *M*_x_O⋅*m*B_2_O_3_ with *m* > 1, there is *n* = *m* − 1 relationship (where *n* = n_∆_/n_t_);
(9) As an exclusion for 2D and 3D highly condensed polyanions, the coordination numbers of oxygen atoms (relative to boron) can be equal to one or three (in cubic boracite even to four).

The first (primary) classification level, successfully used in inorganic chemistry and mineralogy, is known to be based on the type of the anion-forming element (sulphides, halides, silicates, borates, phosphates, etc.). This reflects the characteristic common features of all classes of compounds, determined by the position of anion forming elements in the Periodic table of the elements. The second order (sublevel) represents the subdivision of classes and is usually also based on the composition of compounds or on their structure. For silicates, e.g., in their overwhelming majority natural and, therefore, of complex composition, in which it is sometimes difficult to identify the predominant cations, the crystallochemical classification reflecting the functional dependence between the composition and the structure of the anion has proved to be the most expedient. This, however, does not mean that this approach should be used for systematics onto the other classes of compounds as well.

When the main cations and the above nine prerequisites are considered together, it becomes possible to move the structural principle onto a higher rank (level) of the classification scheme. It is because the structure of polyanions, the polymerization degree, the ratio of the BO_3_ triangles number to the amount of tetrahedra are determined, to a considerable extent, by the N-factor and the type of cation.

The first level of the systematics of anhydrous borates can be subdivided by the quantitative composition of anions and polyanions into the following sublevels:

(a) Borates proper (also, there is a sense to divide this very numerous group, having diverse cations, into two subgroups: aI—monocationic or “simple” borates and all-binary and more complex compounds);
(b) Borosilicates;
(c) Boroaluminates;
(d) Boroberyllates;
(e) Borocarbonates;
(f) Boromolybdates and borotungstates.

It is reasonable to carry out the next, second, order (sublevel) of classification by the value of cation charge. Then, inside these subdivisions, compounds can be ranked in accordance with their decreasing N factor, indicating its value and structural type, if the structure has been studied. Therefore, the third level is structural. Moreover, finally, the fourth order of this scheme should as far as possible represent the change in the type and size of cations having the same charge. It is also expedient to single out the isostructural and isomorphic series, that especially characteristic for borates with isolated BO_3_–triangles.

An example of the scheme of classification of high-temperature anhydrous borates is given in Table 5 and Table 6, where the example of systematics of monocationic mono- and bivalve anhydrous metal compounds is shown. This systematics allows to cover all known anhydrous borate compounds, and to develop an understanding of variations of their properties, limits of stability, as well as the possibility of synthesizing new compounds of these series. Following [98], e.g., Li_2_O⋅4B_2_O_3_ borate seems to contain three-dimensional boron-oxygen nets with the ratio of *n* ≥ 1. The polyanions in Na_2_O⋅5B_2_O_3_ and Na_2_O⋅9B_2_O_3_ are most probably three-dimensional with n equal to 4 and 8, K_2_O⋅3B_2_O_3_ possibly also contains three-dimensional nets of triborate groups with *n* = 2. The structure of polyanions in Rb-borates is close to that in the corresponding potassium compounds. The structures of Cs_2_O⋅4B_2_O_3_ and Cs_2_O⋅5B_2_O_3_ are evidently also three-dimensional with *n* = 3 and 4.

## 8. Structural Aspects of Acid-Base Properties

Understanding of growth kinetics and mechanism of borate crystals from melts and fluxed melts is still a problem and leads to deterioration in crystal quality. Thus, it is useful to consider a correlation between the polymerization of anions in the structures of anhydrous borates and their derivatives in order to explain the capability of these inorganic polymers to crystallize.

There were some attempts to estimate acid-base properties for oxide compounds, both solids and melts. The most popular of them is the Lux-Flood’s acid-base theory [99]. This concept seems to be more effective for assessment of the acid-base characteristics of anhydrous borates finding of promising solvents for the flux growth of high-temperature borate crystals. According to the Lewis-Lux’s equation: Acid + O^2−^ ↔ Base, the acid-base parameters of melts depend on the oxygen activity, thus, B_2_O_3_ + O^2−^ = B_2_O_5_^4−^, which means that the pyroborate anion (2∆) has higher oxygen activity in comparison with boron trioxide increasing its base component.

Therefore, the activity of O^2−^, and, correspondingly, the reactivity of boron-containing melts decreases with an increase in the N_B_/N_O_ ratio, and a tendency to glass formation is observed due to the features of their structure, for which the B–O bond energy is 519 kJ/mol [100]. Since atoms and other particles in such viscous systems move slowly, the glasses obtained by rapid quenching retain pronounced traces of frozen processes.

**Figure 9:**
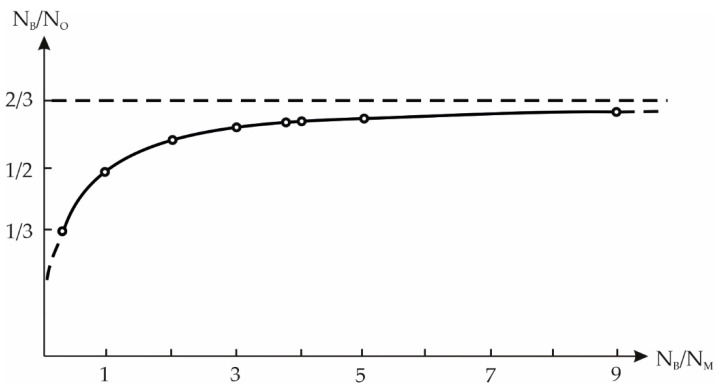
Dependence of anion polymerization (N_B_/N_O_, i.e., ratio of metal atoms number to the ratio of boron atoms number, referred to as N-factor) on N_B_/N*_M_*) in anhydrous alkali borate metal structures.

Taking into consideration the above borate classification and the Lux-Flood’s concept, it is intuitively obvious that the simplest way to assess quantitatively acid-base properties of the anhydrous borates is to estimate the dependence of polymerization of anions in the borate structures on the sizes and valences of cations, and also on the N-factor (Figure 9). From the crystallochemical point of view, it can see that the increase of the N-factor increases the anion polymerization and the N_B_/N_O_ ratio. This leads to a decrease in the oxygen activity factor and simultaneously to an increase in the acid component of these compounds. Also, the value of *n* = n_∆_/n_t_, i.e., the ratio of the number of BO_3_–triangles to BO_4_–tetrahedra in the structures of compounds increases.

## 9. Summary

This review is an alternative approach by the authors to present the structural aspects of high-temperature anhydrous borates in the way of synthesis and growth of crystals of new technologically attractive materials from this numerous family of borates. They can be described by only three types according to the level of complexity of structural units: (1) BO_3_–triangles (∆) and BO_4_–tetrahedra (t) as fundamental (elementary) structural units (*FSU*) constituting the anions of all borates (only triangles, only tetrahedra or both the triangles and tetrahedra); (2) the second level of structural units is represented by combined basic units (*CSU*) which usually built up of several *FSU* (from 2 to 5) joined by sharing common O atoms occurring in many structures; (3) the third type of borate structural units corresponds to complete radicals of polyanions (*CRP*) which constructed of 2–9 *FSU*, i.e., with a composition equal or aliquot to the anionic portions of the compound structural formulas. With a decrease in the *N* = N*_M_*/N_B_ ratio, i.e., N-factor, as well as with an increase in the cation size (though to a smaller extent), the anion polymerization degree and the *n* = n_∆_/n_t_ number (at *N* < 1) regularly increase. An increase in the cation charge causes the reverse tendency. It facilitates the attenuation of the polycondensation of BO_3_–triangles BO_4_–tetrahedra. In borates with highly charged cations, the boron atoms prefer tetrahedral coordination. Highly charged cations are, however, capable to form around themselves rigid coordination polyhedra, usually making up the basis of the structure. Stability of the boron-oxygen anion here loses its decisive importance. This encourages the formation of borate structures with isolated both BO_3_–triangles and BO_4_–tetrahedra having comparatively large charges, −3 and −5 respectively. Tendency to polymerization and, therefore, to more acidic properties makes it possible to forecast new phase systems for the synthesis of predicted borate structures. A new approach to borates classification is proposed, and an improved systematics of anhydrous compounds has been performed. The place of a borate in this scheme, including those with an unsolved structure, characterizes to a certain extent its structure and properties.

## Figures and Tables

**Table 1:** Structural motifs based on the [BO_2_]*_n_* metaborate radical.

Type of Metaborate Anion	Composition of [BO_2_]*_n_* Polyanion	Compound
Isolated rings	[B_3_^∆^O_6_] = [B_3_O_6_]	α-Na_2_O·B_2_O_3_ K_2_O·B_2_O_3_ Rb_2_O·B_2_O_3_ Cs_2_O·B_2_O_3_ β-BaO·B_2_O_3_
Chain (one-dimensional, 1D)	[B_2_^∆^O_4_]_1D_ = [B_2_O_4_]_1D_	α-Li_2_O·B_2_O_3_ CaO·B_2_O_3_ (I) SrO·B_2_O_3_ (I)
	[(B^∆^O_2_)_2_B_2_^t^O_4_]_1D_ = [B_4_O_8_]_1D_	Calciborite CaO·B_2_O_3_ (II)
	[(B^∆^O_2_)_4_B^t^O_2_]_1D_ = [B_6_O_12_]_1D_	*Ln*_2_O_3_·3B_2_O_3_ (*Ln* = La, Ce, Pr, Nd, Sm, Eu, Gd, Tb)
	[(B^∆^O_2_)_2_Al_2_^t^O_4_]_(1D)_ = [B_2_Al_2_O_8_]_1D_	2CaO·Al_2_O_3_ B_2_O_3_
	[(BO_2_)_2_Al_2_^t^O_4_]_1D_ = [B_2_Al_2_O_8_]_1D_	2SrO·B_2_O_3_·Al_2_O_3_
	[B_3_^t^O_6_]_(1D)_ = [B_3_O_6_]_1D_ (exc. Si)	Stillwellite Ce_2_O_3_·B_2_O_3_·2SiO_2_
	[B_2_^∆^O_4_](D) = [B_2_O_4_]_1D_ (exc. Mo, W)	La_2_O_3_·B_2_O_3_·2MoO_3_ La_2_O_3_·B_2_O_3_·2WO_3_
Ribbon (one-dimensional, 1D)	[(B^∆^O_2_)_4_(Al^t^O_2_)_2_]_1D_ = [B_4_Al_2_O_12_]_1D_ [(B^∆^O_2_)_4_(Ga^t^O_2_)_2_]_1D_ = [B_4_Ga_2_O_12_]_1D_	3Li_2_O·Al_2_O_3_·2B_2_O_3_ 3Li_2_O·Ga_2_O_3_·2B_2_O_3_
Layer (two-dimensional, 2D)	[(B^∆^O_2_)_4_B_6_^t^O_12_]_2D_ = [B_10_O_20_]_2D_	*Ln*_2_O_3_·2CoO·5B_2_O_3_ (*Ln* = La-Nd, Sm-Ho)
Framework (three-dimensional, 3D)	[B^t^O_2_]_3D_ = [BO_2_]_3D_	γ-Li_2_O·B_2_O_3_
	[B_3_^t^O_6_]_3D_ = [B_3_O_6_]_3D_	CaO·B_2_O_3_ (II) SrO·B_2_O_3_ (II)
	[B_3_^t^O_6_]_3D_ = [B_3_O_6_]_3D_	CuO·B_2_O_3_
	[B_6_^t^O_12_]_3D_ = [B_6_O_12_]_3D_	4ZnO·3B_2_O_3_
	[(B_6_^∆^O_2.5_)(B^∆^O_1.5_)B_4_O_8_]_3D_ = [B_6_O_12_]_3D_	CaO·B_2_O_3_ (III) SrO·B_2_O_3_ (III)
	[(B_2_^t^O_4_)(Si_2_O_4_)]_(3D)_ = [Si_2_B_2_O_8_]_3D_	Danburite—CaO·B_2_O_3_·2SiO_2_

**Table 2:** Polyanions with [B_4_O_7_]*_n_* and [B_8_O_14_]*_n_* radicals.

Type of polyanion	Composition of B_4_O_7_ Based Polyanion	Compound
Layers (two-dimensional, 2D)	[(B_2_^∆^B^t^O_5.5_)(B_3_^∆^B_2_^t^O_8.5_)]_2D_ = [B_8_O_14_]_2D_	α-Na_2_O·2B_2_O_3_
Frameworks (three- dimensional, 3D)	[B_2_^∆^B_2_^t^O_7_]_3D_ = [B_4_O_7_]_3D_	LiO·2B_2_O_3_
	[B_2_^∆^B_2_^t^O_7_]_2(3D)_ = [B_4_O_7_]_2(3D)_	MgO·2B_2_O_3_, MnO·2B_2_O_3_, ZnO·2B_2_O_3_, CdO·2B_2_O_3_
	[(B_2_^∆^B_2_^t^O_7_)(B^∆^B_2_^t^O_5.5_)(B^∆^O_1.5_)]_3D_ = [B_8_O_14_]_3D_	K_2_O·2B_2_O_3_
	[(B_2_^∆^B_2_^t^O_7_)(B_2_^∆^B^t^O_5_)(B^t^O_2_)]_3D_ = [B_8_O_14_]_3D_	CaO·2B_2_O_3_
	[B_4_^t^O_7_]_3D_ = [B_4_O_7_]_3D_	SrO·2B_2_O_3_, PbO·2B_2_O_3_
	[(B_3_^∆^B_2_^t^O_8.5_)(B^∆^B_2_^t^O_5.5_)]_3D_ = [B_8_O_14_]_3D_	BaO·2B_2_O_3_

**Table 3:** Polyanions based on [B_3_O_5_]*_n_* and [B_8_O_13_]*_n_* radicals.

Radical	Type of Polyanion	Composition B_3_O_5_ and B_8_O_13_ Based Polyanions	Compound
B_3_O_5_	Layers (two-dimensional, 2D)	[(B_4_^∆^B^t^O_8_)(B_2_^∆^B^t^O_5_)(B^t^O_2_)]_2(2D)_ = [B_9_O_15_]_2(2D)_	β-Na_2_O·3B_2_O_3_
	Frameworks (three-dimensional,3D)	[(B_4_^∆^B^t^O_8_)(B_2_^∆^B_2_^t^O_7_)]_2(3D)_ = [B_9_O_15_]_2(3D)_	α-Na_2_O·3B_2_O_3_
		[B_2_^∆^B^t^O_5_]_3D_ = [B_3_O_5_]_3D_	Cs_2_O·3B_2_O_3_
B_8_O_13_	Frameworks (three-dimensional, 3D)	[(B_4_^∆^B^t^O_8_)(B_2_^∆^B^t^O_5_)]_2(3D)_ = [B_8_O_13_]_2(3D)_	α-Na_2_O·4B_2_O_3_
		[(B_4_^∆^B^t^O_8_)(B_2_^∆^B^t^O_5_)]_2(3D)_ = [B_8_O_13_]_2(3D)_	0.6Ag_2_O·0.4Na_2_O·4B_2_O_3_; BaO·4B_2_O_3_

**Table 4:** Structural formulas of monocationic polyborates.

Formula in Oxides (Bulk Composition)	Structural Formula		
	Complete		Abbreviated
α-Li_2_O·B_2_O_3_	α-Li_2_[B^∆^_2_O_4_]_1D_	α-Li_2_[B^△^_2_O_4_]_1D_	α-Li_2_[B_2_O_4_]
γ-Li_2_O·B_2_O_3_	γ-Li[B^t^O_2_]_3D_	γ-Li[B^t^O_2_]_3D_	γ-Li[BO_2_]
γ-3Li_2_O·7B_2_O_3_·2LiCl	γ-Li_4_Cl[(B^∆^_3_B^t^_3_O_10.5_)(B^∆^O_1.5_)]_3D_	γ-Li_4_Cl[B^△^_4_B^t^_3_O_12_]_3D_	γ-Li_4_Cl[B_7_O_12_]
Li_2_O·2B_2_O_3_	Li_2_[(B^∆^_2_B^t^_2_O_7_)]_3D_	Li_2_[B^△^_2_B^t^_2_O_7_]_3D_	Li_2_[B_4_O_7_]
*M*^I^_2_O·B_2_O_3_ (M^I^ = Na-Cs)	*M*^I^_3_[(B^∆^_3_O_6_)]	*M*^I^_3_[B^△^_3_O_6_]	*M*^I^_3_[B_3_O_6_]
α-Na_2_O·2B_2_O_3_	α-Na_4_[(B^∆^_2_B^t^O_5.5_)(B^∆^_3_B^t^_2_O_8.5_)]_2D_	α-Na_4_[B^△^_5_B^t^_3_O_14_]_2D_	α-Na_4_[B_8_O_14_]
α-Na_2_O·3B_2_O_3_	α-Na_6_[(B_2_B^t^_2_O_7_)(B^∆^_4_B^t^O_8_)]_2(3D)_	α-Na_6_[B^△^_6_B^t^_3_O_15_]_2(3D)_	α-Na_6_[B_9_O_15_]_2_
β-Na_2_O·3B_2_O_3_	β-Na_6_[(B^∆^_2_B^t^O_5_)(B^∆^_4_B^t^O_8_)(B^t^O_2_)]_2(2D)_	β-Na_6_[B^△^_6_B^t^_3_O_15_]_2(2D)_	β-Na_6_[B_9_O_15_]_2_
α-Na_2_O·4B_2_O_3_	α-Na_4_[(B^∆^_2_B^t^O_5_)(B^∆^_4_B^t^O_8_)]_2(3D)_	α-Na_4_[B^△^_6_B^t^_2_O_13_]_2(3D)_	α-Na_4_[B_8_O_13_]_2_
K_2_O·2B_2_O_3_	K_4_[(B^∆^B^t^_2_O_5.5_)(B^∆^_2_B^t^_2_O_7_)(B^∆^O_1.5_)]_3D_	K_4_[B^△^_4_B^t^_4_O_14_]_3D_	K_4_[B_8_O_14_]
5K_2_O·19B_2_O_3_	K_2.5_[(B^∆^_2_B^t^O_5_)(B^∆^_4_B^t^O_8_)(B^∆^O_1.5_)(B^t^_0.5_O)]_3D_	K_5_[B^△^_14_B^t^_5_O_31_]_3D_	K_5_[B_19_O_31_]
α-K_2_O·5B_2_O_3_	α-K_2_[(B^∆^_4_B^t^O_8_)]_2(3D)_	α-K_2_[B^△^_4_B^t^O_8_]_2(3D)_	α-K_2_[B_5_O_8_]_2_
β-*M*^I^_2_O·5B_2_O_3_ (*M*^I^ = K, Rb)	β-*M*^I^_2_[(B^∆^_4_B^t^O_8_)]_2(3D)_	β-*M*^I^_2_[B^△^_4_B^t^O_8_]_2(3D)_	β-*M*^I^_2_[B_5_O_8_]_2_
Cs_2_O·3B_2_O_3_	Cs[(B^∆^_2_B^t^O_5_)]_3D_	Cs[B^△^_2_B^t^O_5_]_3D_	Cs[B_3_O_5_]
Cs_2_O·9B_2_O_3_	Cs_2_[(B^∆^_3_O_4.5_)_2_(B^∆^_2_B^t^O_5_)]_2(3D)_	Cs_2_[B^△^_8_B^t^O_14_]_2(3D)_	Cs_2_[B_9_O_14_]_2_
0.6Ag_2_O·0.4Na_2_O·4B_2_O_3_	Ag_2.4_Na_1.6_[(B^∆^_2_B^t^O_5_)(B^∆^_4_B^t^O_8_)]_2(3D)_	Ag_2.4_Na_1.6_[B^△^_6_B^t^_2_O_13_]_2(3D)_	Ag_2.4_Na_1.6_[B_8_O_13_]_2_
5*M*^II^O·7B_2_O_3_·*M*^II^*A*^I^_2_—rhomb. and trig. Boracites (*M*^II^ = Mg, Mn, Zn, Cd, Co, Ni, Cu; *A*^I^ = Cl, NO_3_)—rhomb. (*M*^II^ = Mg, Mn, Zn, Fe, Co, Ni, Cu; *A*^I^ = F, Cl)—trig.	*M*_3_^II^*A*^I^[(B^∆^O_1.5_)_2_(B^t^_6_O_11.5_)]_3D_	*M*_3_^II^*A*^I^[B^△^B^t^_6_O_13_]_3D_	*M*_3_^II^*A*^I^[B_7_O_13_]
5*M*^II^O·7B_2_O_3_·*M*^II^*A*^I^_2_—cub. Boracites (*M*^II^ = Mg, Mn, Fe, Ni, Co, Cu; *A*^I^ = Cl, Br, NO_3_)	*M*_3_^II^*A*^I^[(B^t^_7_O_13_)]_3D_	*M*_3_^II^*A*^I^[B^t^_7_O_13_]_3D_	*M*_3_^II^*A*^I^[B_7_O_13_]
*M*^II^O·2B_2_O_3_ (*M*^II^ = Mg, Mn, Zn, Cd)	*M*_2_^II^[(B^∆^_2_B^t^_2_O_7_)]_2(3D)_	*M*_2_^II^[B^∆^_2_B^t^_2_O_7_)]_2(3D)_	*M*_2_^II^[B_4_O_7_)]_2_
*M*^II^O·B_2_O_3_-I (*M*^II^ = Ca, Sr)	*M*^II^[(B^∆^_2_O_4_)]_1D_	*M*^II^[B^∆^_2_O_4_]_1D_	*M*^II^[B_2_O_4_]
CaO·B_2_O_3_-II (calciborate)	Ca_2_[(B^∆^O_2_)_2_B^t^_2_O_4_)]_1D_	Ca_2_[B^∆^_2_B^t^_2_O_4_]_1D_	Ca_2_[B_4_O_8_]
*M*^II^O·B_2_O_3_-III (*M*^II^ = Ca, Sr)	*M*_3_^II^[(B^∆^O_1.5_)(B^∆^O_2_)(B^t^O_2_)(B^t^_3_O_6_)]_3D_	*M*_3_^II^[B^∆^_2_B^t^_4_O_12_]_3D_	*M*_3_^II^[(B_6_O_12_)]
*M*^II^O·B_2_O_3_-IV (*M*^II^ = Ca, Sr)	*M*_3_^II^[(B^t^_3_O_6_)_2_]_3D_	*M*_3_^II^[(B^t^_3_O_6_)_2_]_3D_	*M*_3_^II^[(B^t^_3_O_6_)_2_]
2CaO·3B_2_O_3_	Ca_2_[(B^∆^_2_B^t^_3_O_9_)_2_(B^t^O_2_)]_3D_	Ca_2_[B^∆^_2_B^t^_4_O_11_]_3D_	Ca_2_[B_6_O_11_]
CaO·2B_2_O_3_-II	Ca_2_[(B^∆^_2_B^t^O_5_)(B^∆^_2_B^t^_2_O_7_)(B^t^O_2_)]_3D_	Ca_2_[B^∆^_4_B^t^_4_O_14_]_3D_	Ca_2_[B_8_O_14_]
*M*^II^O·2B_2_O_3_ (M^II^ = Sr, Po)	*M*^II^[(B^t^_4_O_7_)]_3D_	*M*^II^[B^t^_4_O_7_]_3D_	*M*^II^[B_4_O_7_]
β-BaO·B_2_O_3_	Ba_3_[(B^∆^_3_O_6_)_2_]	Ba_3_[(B^∆^_3_O_6_)_2_]	Ba_3_[(B_3_O_6_)_2_]
BaO·2B_2_O_3_	Ba_2_[(B^∆^B^t^_2_O_5.5_)(B^∆^_3_B^t^_2_O_8.5_)]_3D_	Ba_2_[B^∆^_4_B^t^_4_O_14_]_3D_	Ba_2_[B_8_O_14_]
BaO·4B_2_O_3_	Ba_2_[(B^∆^_2_B^t^O_5_)(B^∆^_4_B^t^O_8_)]_2(3D)_	Ba_2_[B^∆^_6_B^t^_2_O_13_]_2(3D)_	Ba_2_[B_8_O_13_]_2_
4ZnO·3B_2_O_3_	Zn_4_O[(B^t^_6_O_12_)]_3D_	Zn_4_O[B^t^_6_O_12_]_3D_	Zn_4_O[B_6_O_12_]
CuO·B_2_O_3_	Cu_3_[(B^t^_3_O_6_)_2_]_3D_	Cu_3_[(B^t^_3_O_6_)_2_]_3D_	Cu_3_[(B_3_O_6_)_2_]
5Al_2_O_3_·B_2_O_3_	Al_2_[(Al^t^_3_O_6_)(B^∆^O_3_)]_3D_	Al_2_[Al^t^_3_B^∆^O_9_]_3D_	Al_2_[Al_3_BO_9_]
*R*_2_O_3_·3B_2_O_3_ (*R* = La-Tb)	*R*_2_[(B^∆^_2_B^t^_2_O_8_)(B^∆^O_2_)_2_]_1D_	*R*_2_[B^∆^_4_B^t^_2_O_12_]_1D_	*R*_2_[B_6_O_12_]

M^I^—monovalent metal; M^II^—divalent metal.

**Table 5:** Classification scheme of anhydrous borates.

By the Composition of Anion Formers		By Cation Valency		By Cation Type (and Size)	By the Value of N Factor (*N* = N*_M_*/N_B_)				
					*N* > 1	*N* = 1	*n* = n_∆_/n_t_ = *m* − 1, where *m*—coeff. from *M*_x_O·*m*B_2_O_3_ *		
							1 < *N* ≤ 1/2	1/2 < *N* ≤1/3	*N* < 1/3
Borates	Monocationic (simple)	Monovalent	a	Li ↓ Cs	Orthoborates with isolated BO_3_ triangles	Metaborates, insular and chainlike **	Polyborates, 3D and seldom - layered	Polyborates, 3D and seldom - layered	Polyborates, 3D
			b	Ag Tl	Same	0	0	0	Same
		Divalent	a	Be ↓ Ba	Same	Pyroborates	Metaborates, insular and chainlike**	Polyborates, 3D	Same
			b	Zn, Cd, Mn, Fe, Co, Ni, Cu, Pb	Same	Same	Metaborates, 3D	Same	Same
		Trivalent	a	Al	Orthoborates with BO_4_ tetrahedra	Orthoborates with BO_3_ triangles	-	-	-
			b	Sc, Ti, V, Cr, Ga, In	-	Same	-	-	-
			c	Fe	Orthoborates with BO_4_ tetrahedra	Same	-	-	-
			d	Y, La-Nd, Sm-Yb	0	Same	-	Metaborates, chainlike	-
			e	Bi	Orthoborates with BO_3_ triangles	-	-	0	0
		Tetravalent		Th	-	-	0	-	-
		Pentavalent		P, As, Ta, Nb	-	Orthoborates with BO_3_ triangles	-	-	-
1		2		3	4	5	6	7	8
Borates	Binary and more complex	*M* ^+^ *M* ^2+^			Orthoborates with BO_3_ triangles	-	-	-	-
		*M* ^+^ *M* ^3+^			Same	0	0	-	-
		*M* ^+^ *M* ^5+^			Same	Pyroborates	-	-	-
		*M* ^2+^ *M* ^2+^			-	Same	-	-	-
		*M* ^2+^ *M* ^3+^			Orthoborates with BO_3_ triangles and BO_4_ tetrahedra	-	Metaborates, layered (t)	Metaborates, layered (∆ + t)	-
		*M* ^2+^ *M* ^4+^			-	Orthoborates with BO_3_ triangles	-	-	-
		*M* ^3+^ *M* ^3+^			-	Orthoborates with BO_3_ triangles	Metaborates with B tetrahedrons	-	-
Boron silicates		*M*^2+^*M*^3+^, *M*^3+^, *M*^2+^			Orthocompounds	Metacompounds, chainlike	-	-	Polycompounds, 3D
Boron aluminates		*M*^2+^, *M*^3+^			-	Same	Polycompounds, layered and 3D	-	-
Boron beryllates		*M* ^+^			-	-	Polycompounds, layered	Polycompounds, layered and 3D	-
Boron carbonates		*M* ^2+^ *M* ^3+^			Orthocompounds	-	-	-	-
Boron molybdates and boron tungstanates		*M* *M* ^3+^			-	-	Metacompounds, chainlike	-	-

* Simple mono- and divalent metal borates having polyanions with coordination number of oxygen atoms with respect to boron are equal to two obey this rule. ** 3D γ-LiBO_2_, Ca and Sr metaborates obtained at high pressure are an exception. Note: Symbols “-” and “0” indicate that this compound is unknown (“-”) or it is known but its structure was not solved (“0”).

**Table 6:** Classification of mono- and divalent metal borates.

I. Borates of Monovalent Elements									
		N	Cations						
			Li	Na (Ag)		K	Rb	Cs	Tl
Orthoborates (with isolated B triangles)		5	-	0		-	-	-	-
		3	α-Li_3_[BO_3_] β from – 0	0		-	-	-	Tl_3_[BO_3_]
		5/2	-	0		-	-	-	-
		2	-	0		-	-	-	-
		3/2	0	0		-	-	-	-
Fluoroborates (with isol. BF_4_ tetr.)		1	-	Na[BF_4_]		K[BF_4_]	Rb[BF_4_]	Cs[BF_4_]	Tl[BF_4_]
Metaborates (ins. with boroxol gr.) *		1	α-Li_2_[B_2_O_4_]_1D_ γ-Li[BO_2_]_3D_	α-Na_3_[B_3_O_6_] β phase – 0		K_3_[B_3_O_6_]	α from - 0	Cs_3_[B_3_O_6_]	0
Polyborates (skeletal, sometimes compounds, layered with diborate – D, triborate – T, pentaborate – P, boroxol – B, ditriborate – DT, dipentaborate – DP rings and additional B triangles and tetrahedrons; *n* = n_∆_/n_t_ = *m* − 1)		2/3	-	0		-	-	-	-
		4/7	α and β Cl, Br and I “boracites” – 0 γ-Li_4_Cl[B_7_O_12_]_3D_	-		-	-	-	-
		1/2	Li_4_[B_4_O_7_]_2(3D)_2(D)	(α)-Na_4_[B_8_O_14_]_2D_ ** (T+DP) β and γ phase - 0		K_4_[B_8_O_14_]_3D_	0	0	0
		2/5	0	0		-	-	-	-
		1/3	0	α-Na_6_[B_9_O_15_]_2(3D)_ 2(D + P) β-Na_6_[B_9_O_15_]_2(2D)_ 2(P + T + t) γ-form – 0		0	0	Cs[B_3_O_5_]_(3D)_ (T)	0
		5/19	-	-		K_5_[B_19_O_31_]_3D_	-	-	-
		1/4	0	α-(Na,Ag)_4_[B_8_O_13_]_2(3D)_ 2(T + P) β modification – 0		0	0	0	0
		1/5		α, β and γ phases – 0		α-K_2_[B_5_O_8_]_2(3D)_ 2(P) β-K_2_[B_5_O_8_]_2(3D)_ 2(P), γ phase - 0	α phase – 0 β-Rb_2_[B_5_O_8_]_2(3D)_ 2(P)	α, β, γ phases - 0	0
		1/9	0	α, β, and γ phases – 0		0	0	Cs_2_[B_9_O_14_]_2(3D)_ 2(B + T) β phase	-
**II. Borates with Cations of Transition Metals**									
	**N**	**Cations**							
		**Zn**	**Cd**	**Mn**	**Fe**	**Co**	**Ni**	**Cu**	**Pb**
Orthoborates (with isolated BO_3_ triangles)	3	-	-	Wiserite Mn_3_[BO_3_]F_3_	-	-	-	-	-
	2	-	-	Mn_2_[BO_3_]F	-	-	-	-	0 (α and β forms)
	3/2	α-Zn_3_[BO_3_]_2_	0 (α and β forms)	Jimboite Mn_3_[BO_3_]_2_	-	0	Ni_3_[BO_3_]_2_	0	-
	5/4	0 (α and β forms)	-	-	-	-	-	-	-
Pyroborates (with isol.2)	1	-	Cd_2_[B_2_O_5_]	Mn_2_[B_2_O_5_]	Fe_2_[B_2_O_5_]	Co_2_[B_2_O_5_]	0	-	0 (α and β forms)
Metaborates (skeletal)	1/2	0 (α and β forms) Zn_4_O[B_6_O_12_] * (t)	-	0	0	0	0	Cu_3_[(B_3_O_6_)_2_] (t)	0
Polyborates (skeletal)	3/7	cub., rhomb. and trig. F, Cl, Br, I and NO_3_ “boracites”	Cl, Br, I and NO_3_ “boracites”	cub., rhomb. and trig. F, Cl, Br, I and NO_3_ “boracites”	cub. and trig. F, Cl, Br, I and NO_3_ “boracites”	cub., rhomb. and trig. F, Cl, Br, I and NO_3_ “boracites”	cub. and rhomb. Cl, Br, I and NO_3_ “boracites”	cub. and rhomb. Cl, Br, I and NO_3_ “boracites”	-
	1/3	-	0	-	-	-	-	-	-
	1/4	Zn_2_[B_4_O_7_]_2_ 2D	Cd_2_[B_4_O_7_]_2_ 2D	Mn_2_[B_4_O_7_]_2_ 2D	-	-	-	-	Pb[B_4_O_7_] (t)
	1/6	0	-	0	-	-	-	-	-

* 3D γ-LiBO_2_ obtained at high pressure is an exception. ** By its *n* = 5/3 number is an exception; in a part of O atoms coordination number with respect to boron is equal to 1.
